# The H2 robotic exoskeleton for gait rehabilitation after stroke: early findings from a clinical study

**DOI:** 10.1186/s12984-015-0048-y

**Published:** 2015-06-17

**Authors:** Magdo Bortole, Anusha Venkatakrishnan, Fangshi Zhu, Juan C Moreno, Gerard E Francisco, Jose L Pons, Jose L Contreras-Vidal

**Affiliations:** Neural Rehabilitation Group, Cajal Institute, Spanish Research Council, Av. Doctor Arce 37, Madrid, 28002 Spain; Noninvasive Brain-Machine Interface Systems Laboratory, Department of Electrical and Computer Engineering, University of Houston, Houston, 77204-4005 USA; TIRR Memorial Hermann and Department of PM&R, University of Texas Health Sciences Center, 1333 Moursund Street, Houston, 77030 USA; Currently at Palo Alto Research Center, a Xerox company, Palo Alto, CA 94304 USA

**Keywords:** Exoskeleton, Gait, Rehabilitation, Lower limb, Stroke

## Abstract

**Background:**

Stroke significantly affects thousands of individuals annually, leading to considerable physical impairment and functional disability. Gait is one of the most important activities of daily living affected in stroke survivors. Recent technological developments in powered robotics exoskeletons can create powerful adjunctive tools for rehabilitation and potentially accelerate functional recovery. Here, we present the development and evaluation of a novel lower limb robotic exoskeleton, namely H2 (Technaid S.L., Spain), for gait rehabilitation in stroke survivors.

**Methods:**

H2 has six actuated joints and is designed to allow intensive overground gait training. An assistive gait control algorithm was developed to create a force field along a desired trajectory, only applying torque when patients deviate from the prescribed movement pattern. The device was evaluated in 3 hemiparetic stroke patients across 4 weeks of training per individual (approximately 12 sessions). The study was approved by the Institutional Review Board at the University of Houston. The main objective of this initial pre-clinical study was to evaluate the safety and usability of the exoskeleton. A Likert scale was used to measure patient’s perception about the easy of use of the device.

**Results:**

Three stroke patients completed the study. The training was well tolerated and no adverse events occurred. Early findings demonstrate that H2 appears to be safe and easy to use in the participants of this study. The overground training environment employed as a means to enhance active patient engagement proved to be challenging and exciting for patients. These results are promising and encourage future rehabilitation training with a larger cohort of patients.

**Conclusions:**

The developed exoskeleton enables longitudinal overground training of walking in hemiparetic patients after stroke. The system is robust and safe when applied to assist a stroke patient performing an overground walking task. Such device opens the opportunity to study means to optimize a rehabilitation treatment that can be customized for individuals.

**Trial registration:** This study was registered at ClinicalTrials.gov (https://clinicaltrials.gov/show/NCT02114450).

## Introduction

Stroke is the main cause of serious long-term disability worldwide [1] and gait is one of the most important activities affected in stroke survivors. In United States alone, about 800.000 people have a new or recurrent stroke every year [2] and most stroke victims experience significant sensory-motor impairments and require rehabilitation to achieve functional independence. In this context, hemiparesis is a manifestation of stroke that affects the contralesional side of the body, and commonly impacts gait [3, 4].

Task-oriented repetitive movements can improve muscular strength and movement coordination in patients with impairments due to neurological disorders such hemiparesis. Gait training on a treadmill, sometimes requiring manual assistance by therapists, is widely used in clinical practice for stroke rehabilitation [5, 6]. Nevertheless, this technique has some limitations because the manual assistance can lead to poor coordination and synchronization of movements in both legs and fatigue of therapist can limit the efficacy of the therapy.

Systematic reviews have considered the benefit of traditional gait training paradigms in combination with high-technology, intensive rehabilitation approaches [7–10]. Compared with conventional therapy, robotic rehabilitation can deliver highly controlled repetitive and intensive training and reduce the burden of clinical staff, besides providing a quantitative assessment of motion and forces. The usage of robotic interventions in training tasks is expected to augment plasticity and improve recovery. Through these applications, patients will benefit as they will be able to recover at a faster rate, thus enabling them to resume their daily activities sooner and returning to functional independence.

Body weight support with treadmill training machines, such as Lokomat [11], ALEX [12] and LOPES [13], have been used for gait training in stroke victims. For more effective results in the rehabilitation process, it is known that the patient’s involvement and participation in voluntary movement of affected limbs is critical [14]. Stationary devices such as GaitTrainer [15] and HapticWalker [16] have shown to facilitate the user’s cognitive engagement and patient compliance during locomotor training. However, a wearable robotic exoskeleton like the H2 allows training functional movements in ecologically valid ambulatory conditions. Therefore, cognitive engagement can be further promoted while relevant sensory inputs and central neuronal circuits may become activated under physiological conditions (i.e., overground walking), and lead to potentially important neural regeneration effects.

Ambulatory exoskeletons with control strategies that challenge the user to perform movements in these real-life environments can be more effective to reinstate neuroplasticity and improve motor functions [17]. Hybrid Assistive Limb (HAL) [18] is an ambulatory exoskeleton that has been used for stroke rehabilitation. Two different control strategies are used with HAL, depending on the treatment purpose and user’s capabilities [19]. The main actuation mechanisms are based on surface electromyography (sEMG) signals, which adjust the robot joint torques for assistance depending on the measured muscle activity. The second algorithm reproduces a stored movement pattern based on acceleration and ground contact forces with the exoskeleton.

Safety and usability of HAL have been evaluated in different studies [20–22]. In the study carried out by Maeshima *et al* [21], which comprised 16 stroke patients with severe hemiplegia, the authors conclude that sEMG signals used to provide power assistance can make it difficult for severely hemiplegic patients to perform activities using their own muscles. This could lead to instability, decreasing stride length and walking speed. Also, the availability and quality of sEMG signals can vary from patient to patient. Fragility and installation requirements of electrodes can also be restrictive outside the laboratory [23]. The system based on sEMG signal requires a process of adaptation and adjustment to a specific user that can take up two months, depending of each person [24].

Other ambulatory exoskeletons that have been evaluated for clinical use are ReWalk [25], Vanderbilt exoskeleton [26], Ekso [27] and Kinesis [28]. All these exoskeletons have been evaluated with paraplegic users, except for the Ekso, which is currently being tested in a clinical trial with stroke patients [29]. A recently developed exoskeleton, Walk Assist, is also being tested in stroke patients [30]. However, there are no published reports on the safety and usability of these systems in stroke patients. NASA (National Aeronautics and Space Administration) in partnership with IHMC (Institute for Human and Machine Cognition) have also developed an exoskeleton called X1 [31] with powered hip and knee joints, intended for future use in space as an compact exercise tool for astronauts. This device was tested with 2 healthy subjects and 1 stroke patient in [32]. It was noted that an actuation on ankle joint in the X1 would be very clinically relevant to counteract the foot drop problems in stroke stroke survivors.

The research presented here describes the development and evaluation of a novel robotic exoskeleton for gait rehabilitation in stroke survivors. The design philosophy differs from treadmill devices, since motivation and full engagement of patients are targeted with overground walking in a real environment. The robotic exoskeleton, named H2 (a totally improved version of the exoskeleton described in [33]), has six joints actuated, including hip, knee and ankle on both legs. To the best of our knowledge, no ambulatory exoskeletons used for rehabilitation have the ankle joint actuated. For gait rehabilitation in stroke victims, actuation on ankle is important to target the foot drop problem, which is common in most patients.

Furthermore, in the H2, an impedance controller creates a force field around a desired joint trajectory to assist patients, only applying the required joint torque to assist completing the gait movements that patients are unable to do so. Force field control uses the concept of assistance-as-needed [34]. Assisting only when patients need it can lead to better results than fixed repetitive training as it is more personalized to each individual ensuring consistent patient engagement in the training [35].

Moreover, H2 presents an open architecture that allows it to be integrated with other devices or systems. Both wired and wireless communication interfaces are present on the device for this purpose. This feature open means for combined studies, allowing, for instance, integration of H2 with neural interfaces. When associated with exoskeletons, neural interfaces can be used for correlating the aspects of learning during rehabilitation, as well as creating brain-machine interfaces that further engage the patients [36].

Here, we describe the H2 design and control algorithm for gait assistance. The aim of this work is two-fold: to present the development of the exoskeleton and secondly, to perform a preliminary analysis, in a pre-clinical study, of the safety and usability of the H2 robotic exoskeleton when used for gait rehabilitation during 4 weeks of intensive training in 3 participants with post-stroke hemiparesis. Specifically, we wanted to explore the applicability of the system in terms of safety and acceptance by patients with impaired gait. Therefore, this study sought to demonstrate safety and usability in post-stroke hemiparetic patients who could at least walk short distances but at low speeds and depending on external aids. While this population does not represent target clinical users, it helps test the applicability of the device in users with gait deficits, and in whom successful overground walking training with the H2 could provide added benefits (e.g., increased speed, improved intra- and inter-limb coordination during gait, reduced dependence on assistive device etc.)

## Exoskeleton design

H2 is a lower limb exoskeleton designed for rehabilitation of adults between 1.50 and 1.95 m in height, with a maximum body weight of 100 kg, such as stroke patients following neurological insults. It also can be used for gait compensation in patients who have paralysis of the lower limbs following spinal cord injuries. It is conceived for overground gait training in a clinical environment as a bilateral wearable device with six degrees of freedom (DoF), in which hip, knee and ankle are powered joints.

Various criteria informed the mechanical design. As pointed out in previous work [37], an exoskeleton design should be ergonomic, comfortable, lightweight, with a strong structure, adaptable to different users and with safety in mind. In H2, aluminum 7075 is primarily used in the mechanical structure in consideration of mechanical resistance and lightweight. The final device weights about 12 kg including its battery pack. The exoskeleton frame has bilateral uprights for the thigh and the shank, hinged hip, knee and ankles and articulated footplates (distally) and a waist support (proximally). The mechanical structure is designed to allow active and passive movements in the sagittal plane. In the frontal plane, passive movements of about 20 degrees are possible in the hip joint, allowing for turns while walking. The range of motion (ROM) in actuated joints is mechanically limited for safety reasons. The maximum ROM possible across all joints is shown in Table 1. For the ankle, plantarflexion is shown as extension and dorsiflexion as flexion. These values were chosen based on normal gait on healthy subjects [38], also allowing users to perform sit-to-stand and stand-to-sit movements.

**Table 1:** H2 range of motion

	Hip	Knee	Ankle
Flexion	100 °	100 °	20 °
Extension	20 °	3 °	20 °

The length of the thigh and the shank can be adjusted via a mechanism of two telescopic bars that are pushed one inside the other, and are fixed in different positions. The same mechanism is used to change the position of the foot relative to the exoskeleton’s ankle. The size and position of adjustable rounded leg braces carriers with Velcro straps allow for customization to individual requirements. Foam pads are used to minimize pressure against the skin and prevent damage. The exoskeleton supports its own weight through the mechanical frame to the ground, so the users do not feel any extra weight in their lower limbs.

### Actuators

Most types of actuators used in robotics cannot be used in exoskeletons, since for this application high torques are required while operating at higher speeds that most actuators can provide [39]. Main candidates available for use as actuators in exoskeletons are electric, pneumatic, hydraulic and series elastic actuators (SEA). The design and selection of H2 actuators were based on average of torque and power of each joint during normal gait (not pathological) at normal speed [38]. A study of different possible candidates was evaluated. The most relevant criteria to select the actuation technology to drive the human joints were the specific power (ratio of actuator power to actuator weight) and portability.

In this regard, linear hydraulic and pneumatic actuators have high power density, but they usually are bulky and present problems of internal leakage and friction [40]. SEAs have been used in some rehabilitation devices [41], but they still face a common limitation about the spring constant of the elastic element that is fixed. The harmonious coordination of force and position between patient and exoskeleton is difficult between different subjects [42]. The literature suggests that the use of electric motors provide a reduction in power consumption during gait [43]. DC motors meet the criteria of necessary power with a compact and portable solution for wearable devices. Based on that, brushless DC motors coupled to a type Harmonic Drive gearbox were selected.

A 100 W motor (Maxon, EC60 Flat Brushless) is used in the hip, knee and ankle joints. This motor has a rated voltage of 24 VDC and nominal torque of 220 mNm. Furthermore, a gearbox (Harmonic Drive, CSD-20-160-2AGR) is coupled to the motor shaft in order to reduce speed and increase torque. Harmonic Drive gearboxes were selected to reduce the weight and size of the final actuators. A gear ratio of 160:1 gives to each joint a continuous net torque of 35 Nm and peak torques of 180 Nm. According to [11], an average torque of 35 Nm for the hip actuator is presumed to be sufficient for most patients.

### Power supply

Power supply can be one of the most limiting factors for an untethered exoskeleton embodiment. Although the H2 exoskeleton is designed to be used in a clinical setting, a tethered device can lead to some drawbacks when performing overground walking. Thus, the exoskeleton was developed as an autonomous device. Different types of energy sources have been used to power exoskeletons [39]. With improvements in battery technologies over the years, a compact and higher capacity battery pack can provide enough power for running an exoskeleton.

Autonomy also has to do with the performance of the actuators. The developed exoskeleton was designed with high efficiency motors and gearboxes, and state-of-the-art electronic drives with very low dissipation. Additionally, a compact lithium polymer battery pack was specifically designed to power H2. The pack has a nominal voltage of 22.5 VDC and a capacity of 12 Ah. The battery pack is integrated with the mechanical frame and placed at the hip level, providing a comfortable embodiment for the user and no extra weight on the trunk.

### Sensors

The interaction between user and exoskeleton is very important for users’ comfort and safety in a wearable robotic device [44]. Also, when sensors have to be physically placed on human limbs, several issues, specially related to safety, comfort, reliability and donning/doffing process need to be expected and appropriately dealt with.

In terms of physical interface with the human user, H2 is designed in such a way that there are no sensors physically attached to the human. All sensory information comes from sensors placed on the exoskeleton: 6 potentiometers, 18 Hall Effect sensors, 24 strain gauges and 4 foot switches are used to determine parameters such as angular position and velocity, force and interaction torque between user’s limb and exoskeleton.

Each joint is equipped with a precision industrial potentiometer used as an angular position sensor. It exhibits a high linearity and long rotational life. Its stainless steel shaft is coupled to a toothed pulley, and a toothed belt is used to transmit the joint’s motion, to avoid slippage and therefore a loss of absolute reference position.

Strain gauges attached at each link are used as force sensors. These sensors are designed to measure the torque produced by the interaction between the user’s limb and the exoskeleton. The strain gauges are connected in a full Wheatstone bridge to enhance the measurement sensitivity and accuracy. The bridge is excited with 5 VDC and a custom-made electronic circuit balances the bridge for null point measurement, also amplifying the output 500 times. Thus, the output signal is in a range that allows torque measurements from –50 to +50 Nm. This range was chosen based on the maximum torque of the actuators with a safety factor for peak torques. A calibration constant was obtained using a set of calibrated weights and minimized with a least squares algorithm.

**Fig. 1:**
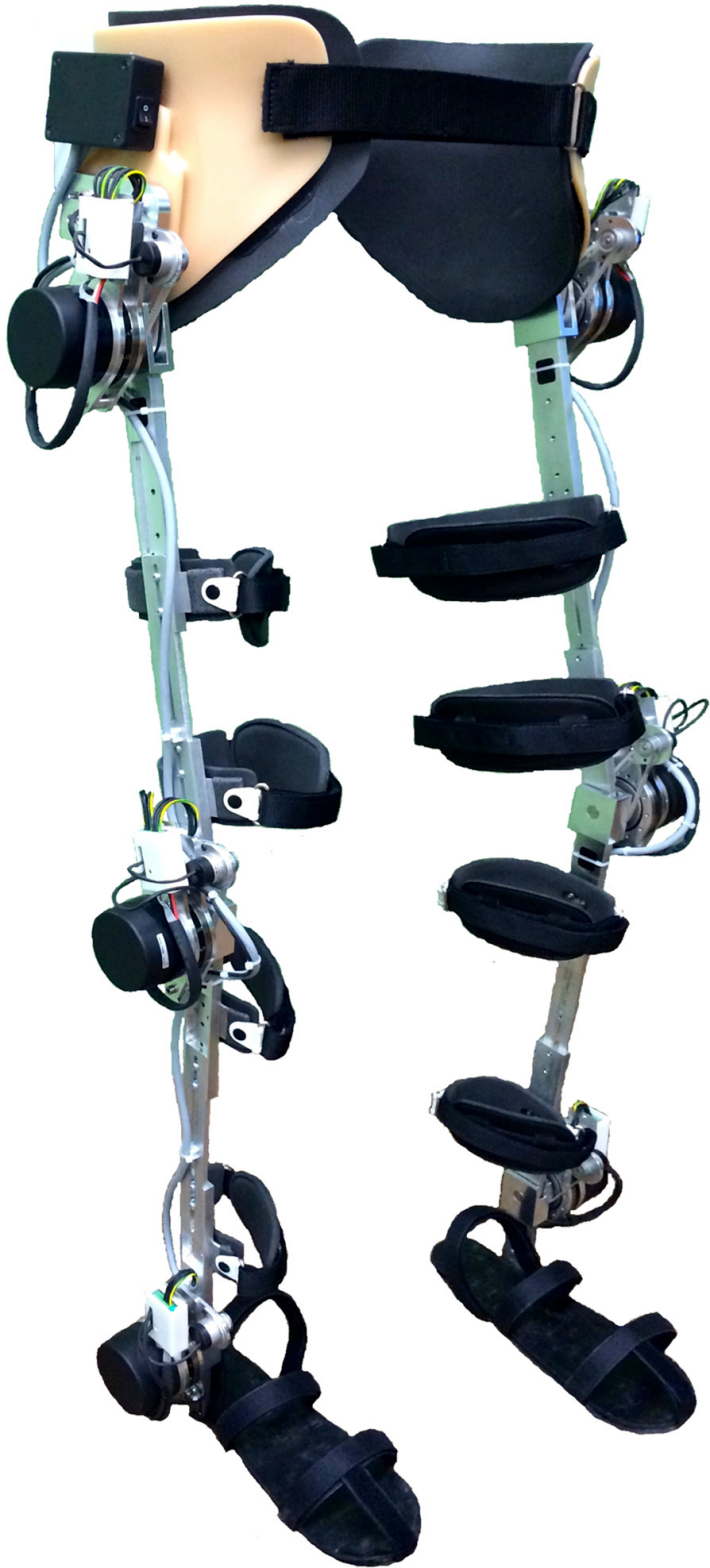
H2 robotic exoskeleton. The six joints are powered by brushless DC motors coupled to Harmonic Drive gearboxes. All sensory information comes from sensors placed on the exoskeleton: 6 potentiometers, 18 Hall Effect sensors, 24 strain gauges and 4 foot switches. A rechargeable battery pack of lithium polymer powers the exoskeleton

The footplate of the exoskeleton is equipped with two foot switches based on resistive sensors, which binary detect the contact between subject’s foot and the ground. These sensors are located under the heel and the toe, and their main goal is to detect the different phases during gait segmentation. The exoskeleton, its actuators and sensors are shown in Fig. 1.

### Control architecture

**Fig. 2:**
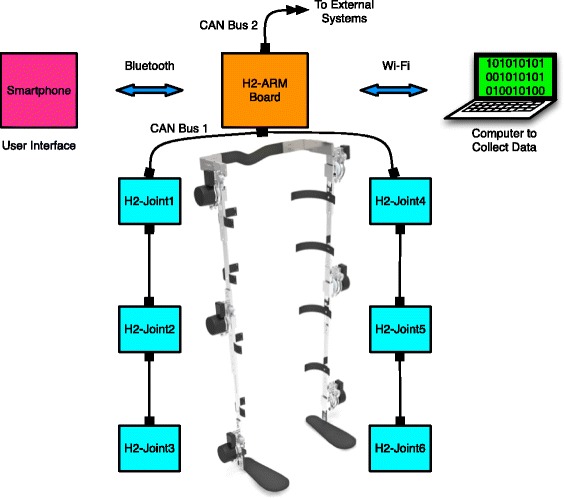
H2 overall control architecture. All sensors in both legs are connected to the H2-Joint1 ∼6 boards that communicate to H2-ARM board through a deterministic real-time network. A Wi-Fi connection is used to capture the kinematic and kinetic data generated in the exoskeleton. A Bluetooth link connects the exoskeleton to a user interface in a smartphone

The control hardware of the exoskeleton is shown in Fig. 2. The main controller is based on a customized electronic board (H2-ARM) designed specifically for real-time control of H2. The small size of H2-ARM board (56 x 44 mm) allows it to be placed on the exoskeleton frame, reducing the bulk, as well as complexity and difficulty of wiring and connections. Moreover, it eliminates the need of a backpack being carried by the user, as most lower limb exoskeletons have.

H2-ARM computational power relies on an STMicroelectronics ARM (Advanced Risk Machine) microcontroller running at 168 MHz. The board has two independent transceiver channels for real time communication: one is used to connect to all six H2-Joint boards, receiving sensory information and commanding the six joint’s actuators; the other channel is intended to connect to external devices.

The board also has two more communication ports, both wireless: Bluetooth and Wi-Fi. Bluetooth communication is intended to connect with a user interface on a smartphone. The user interface is an application that allows physical therapists to adjust certain parameters as needed within the H2 during rehabilitation. More details about the user interface are discussed in the subsequent session. Wi-Fi link is used to send data wirelessly via UDP protocol to a laptop, where the data and information generated in the exoskeleton can be visualized in real time and stored for offline analysis.

Each one of the six joints is equipped with an H2-Joint board (numbered from 1 to 6). Each board is in charge of data acquisition of different joint’s sensors: angular position, interaction torque, joint velocity and foot-ground contact. H2-Joint1 ∼6 contain all the circuitry of the analog filters for each joint sensor and also the amplifiers for the strain gauges. The sensors’ analog output are digitalized by a Digital Signal Processor (DSP) microcontroller after the filtering and amplifying process. A small data packet of six bytes aggregates the sensor’s information on each joint and is sent to H2-ARM controller every one millisecond.

The brushless DC motor’s drives are embedded directly into the H2-Joint ∼6 boards. The drive, developed specifically for this application, is very compact and lightweight, receives digital set points and it is small enough to be mounted directly on the motor side in the exoskeleton’s frame. This approach decreases the amount of electromagnetic noise and the number of wires in the exoskeleton.

A physical communication network that guarantees strict determinism, data collision avoidance and optimized data transfer for small data packets is used to connect the H2-ARM and H2-Joint1 ∼6 boards. The network structure has a deterministic real-time communication based on Control Area Network (CAN) technology running at 1 Mbps. The network allows an unlimited number of nodes (limited only by the electrical load on the bus) and does not require any alteration to add or remove nodes. It is flexible in terms of configuration, automatically avoids data collision and corrects data packets errors in the transmission.

Each communication cycle in the network protocol involves passing a message from the H2-ARM node to all H2-Joint ∼6 nodes in the network. As the message travels on the bus, each H2-Joint ∼6 reads its assigned actuator command data byte (by looking for its own ID number and message byte sequence). Then, each H2-Joint ∼6 returns one message back to H2-ARM node with its locally collected sensor data.

Since the communication cycles occur at a fixed rate (1 kHz) set by the control scheme on H2-ARM, this protocol allows for deterministic control. Also, it provides built-in network error detection as, for every message received, each H2-Joint ∼6 has to return data information to H2-ARM. As a result, H2-ARM has a robust means to determine the integrity of the network and the correct operation of the joint’s actuators. If some failure occurs on the network that cannot be corrected automatically (for instance, a cable disconnection), H2-ARM instantly stops the exoskeleton and shuts off the joint power for safety reasons.

## Control algorithm for gait assistance

Position or trajectory control is a widely implemented robotic strategy [11, 45–48]. In this control mode a position controller guides the patient’s limb to a fixed reference path, while receiving the joint angles as a feedback. For lower limbs, the reference trajectory is a normal gait pattern previously recorded from a healthy subject.

Because the gait pattern differs slightly between individuals, there are some disadvantages to the implementation of a trajectory control based on a pattern of another individual. Recent research efforts are directed at improving therapeutic robot transparency. It is critical that the design of assistive controllers does not hinder the patients’ residual control and provides only the required amount of torque. Thus, newer approaches to actively adjust joint impedance during walking to alter the muscle torque production for a functional purpose, such as modulation of muscle activation, are preferred. When using fixed position tracking controllers in overground exoskeletons, undesired motions, such as those caused by spasms, may cause large actuator forces that could led to unsafe situations. Adding compliance operation to the robotic system can naturally absorb large position errors, thereby compensating for the consequences of these undesired movements. In order to allow for a more compliant operation, we developed an algorithm that takes into account the interaction torque between subject and exoskeleton, in order to produce an adaptive reference for gait assistance.

**Fig. 3:**
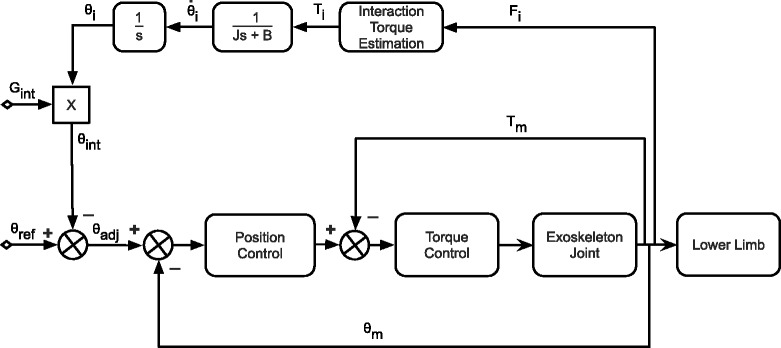
Control scheme for gait assistance. First, the algorithm generates an adaptative reference trajectory. Based on this reference, a force field controller guides the patient limbs, applying the necessary torque to complete gait in each joint independently

Figure 3 represents the algorithm scheme used in H2. The adjusted reference trajectory *θ*_*adj*_ is given by (1) and (2), where *s* is the Laplace operator.

(1) $$ \theta_{adj} = \theta_{ref}-\theta_{int}  $$

(2) $$ \theta_{int} = G_{int}\frac{T_{i}}{Js^{2}+Bs}  $$

*θ*_*ref*_ is a vector containing the recorded angles based on normal gait from a healthy subject and *θ*_*int*_ is the angle related to the interaction torque *T*_*i*_ between exoskeleton and subject’s limb. This angle is estimated using the value of inertia *J* and damping *B* of the exoskeleton frame alone, and it increases or decreases proportionally to the interaction torque between the subject and exoskeleton. If interaction increases, it means that the difference between the trajectory of the subject’s limb and the trajectory of the exoskeleton is higher. Thus, *θ*_*int*_ as given by (2) increases and it is subtracted from *θ*_*ref*_, giving to the position controller a corrected angle. The maximum value that the adapted trajectory can deviate from the recorded trajectory can be adjusted using *G*_*int*_, which is a normalized gain value between 0 and 1, where 0 allows no deviation from reference trajectory. With the user interface, physical therapists can change this gain value ad-hoc for each situation based on the patient’s disability.

The second part of the algorithm is responsible for assisting the patient’s gait based on their disability level. To achieve this, the adapted trajectory feeds a position controller whose output is converted to an input to the torque controller. Consequently, H2 provides an output torque for the actuators, which is proportional to trajectory deviation. The output torque is estimated based on the motor’s electrical current and the gearbox reduction rate. Together, this algorithm creates a force field control that guides patient’s limb in a correct pattern, only assisting patient when he/she deviates from the trajectory. Because all joints on the exoskeleton have their own dedicated electronics and control parameters, each actuator can be independently controlled. This allows the algorithm to generate specific assistance for each joint separately. Especially for hemiparetic stroke patients, who have asymmetric functioning across both lower limbs, this exoskeleton can adapt its functionality in real time based on each individual patient’s needs, without requiring a manual adjustment for each patient.

Furthermore, during training, physical therapists can also adjust the H2’s gait speed, across 10 different possible speeds approximately between 0.5 to 1.8 km/h, to personalize training for each patient. Since H2 adapts the pre-programmed reference based on user’s gait, the absolute final speed is, in some way, user-dependent.

## Methods

In this pilot pre-clinical study, H2 functionality, safety and usability were evaluated in 3 post-stroke hemiparetic users during 4 weeks of gait training. The Institutional Review Board (IRB) at the University of Houston approved all study procedures.

### Subjects

Three eligible male participants with post-stroke left hemiparesis participated in this study after providing informed consent. Demographic data and lesion types for participants are provided in Table 2.

**Table 2:** Demographic data and lesion types for stroke participants

	Patient 1	Patient 2	Patient 3
Age (years)	58	45	43
Height (cm)	192	178	188
Weight (kg)	84	88	99
Stroke onset (months)	60	6	11
Gender	male	male	male
Hemiparesis	left side	left side	left side

### Experimental protocol

**Fig. 4:**
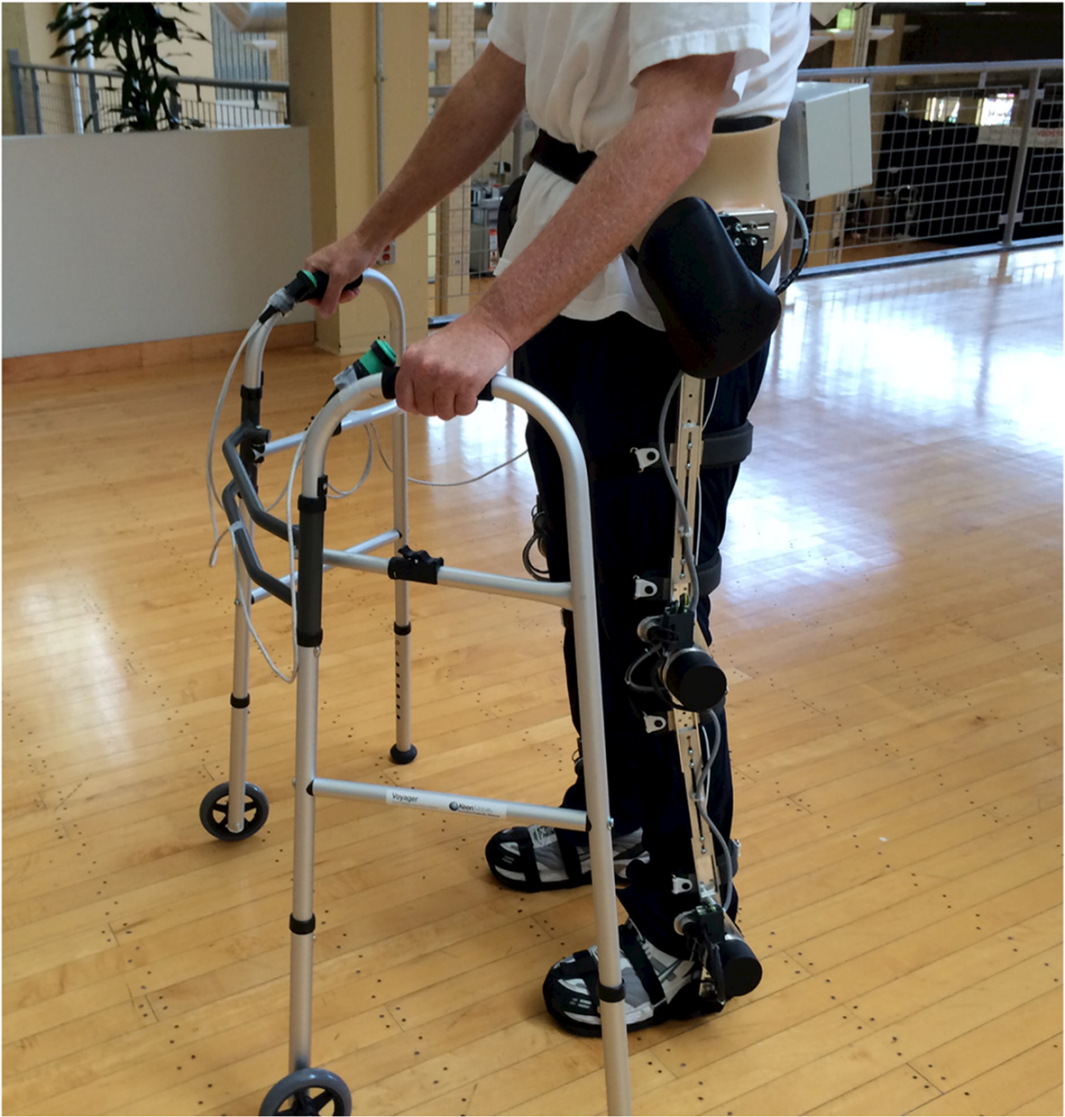
Stroke patient using H2 exoskeleton at the beginning of one training session

In this pilot clinical investigation, the study design consisted of an open-label assignment of participants to H2 robot-assisted gait training. During each training session, subjects were asked to perform an overground walking task guided by the H2 in assist-as-needed mode with a pre-selected gait speed along a 50 m circular or 120 m linear path. After wearing the exoskeleton, patients were instructed to walk as much as they were able and encouraged to take rest breaks as necessary. Figure 4 illustrates a patient using H2 in the beginning of a training session. The gait start and stop process was controlled by the patient using two hand buttons placed on a walker, which was used as a gait assistive device during training.

An experienced physical therapist followed patients during the whole training period. Patients were allowed to change the walking speed in real time during continuous walking from level 1 (slowest) to 10 (fastest) based on their comfort level. Based on patient feedback, the therapist used the smartphone interface to adjust gait speed as necessary. At least two more persons were present during training sessions and followed patients to ensure patient safety. Participants received approximately 12 sessions of training over approximately a 4-week period. All 3 participants were chronic stroke patients and were not receiving any additional gait training or physical therapy during this period of experimental training.

Pre- and post-training (within 2 weeks after training) standard clinical assessments were performed by an independent rater (a second physical therapist that did not participate in the robotic training). The assessment included Berg’s Balance Score, Barthel Index, Functional Gait Index, Fugl-Meyer’s assessment of motor recovery (lower extremity), Timed Up and Go test and 6-minute walk test. These assessments were included to help document any clinically relevant behavioral changes that may occur in response to training with the H2 powered exoskeleton. The study protocol is also registered and available at ClinicalTrials.gov (NCT02114450).

### Data acquisition

Walking angular kinematics, interaction torques and motor torques for left and right hip, knee and ankle joints, together with toe and heel ground contact were sampled at 100 Hz by the H2. All data were transmitted wirelessly over a Wi-Fi link to a laptop via UDP protocol.

## Results

### Robotic exoskeleton intervention

**Fig. 5:**
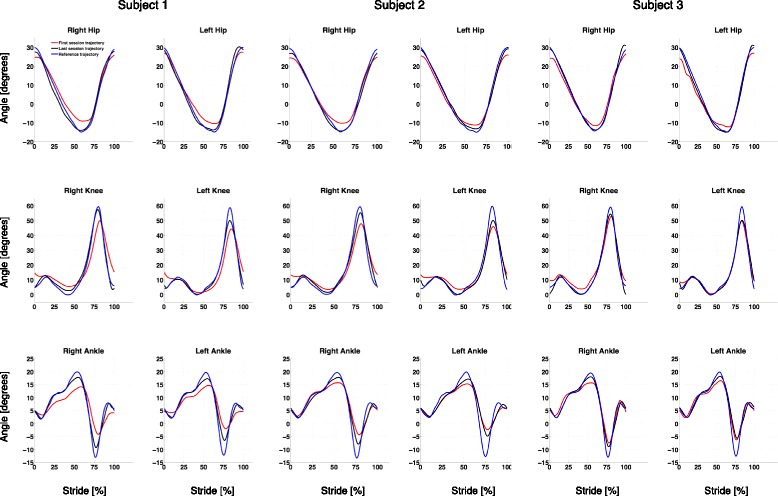
Hip, knee and ankle trajectories performed by all subjects. Blue line is the reference trajectory that patients are guided through by means of a force field. Red line represents the average of all steps performed by subject in the first training session. Black line is the average of all steps performed at last session. Trajectories are represented based on stride length percentage, from heel strike to next heel strike

In this pilot clinical study, the usability and safety of using the H2 for robot-assisted longitudinal gait training in stroke patients has been established. The 3 participants with stroke were able to finish 12 sessions of training over a period of approximately 4 weeks (Subject 2 completed 10 sessions only as he had to miss 2 sessions due to a personal schedule conflict). At the first session, all participants started at the lowest walking speed (0.5 km/h) and were able to increase the gait speed across sessions as training progressed. The deviant gait pattern of the three stroke patients was retrained into a more symmetric pattern during the training time, as seen in the Fig. 5.

**Fig. 6:**
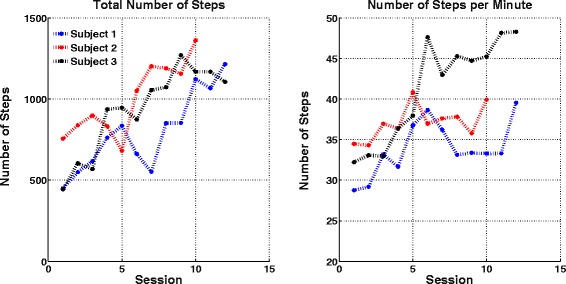
Number of steps performed in all training sessions by all subjects. Although the number of steps and walk speed depends on patient’s conditions and mood on the training day, the overall results clearly show an increase over time

Further, the number of steps walked, a measure indicative of walking distance, increased across sessions for all participants (see Fig. 6). Additionally, the pre- and post-training clinical outcome scores for the 3 participants are presented in Table 3. As shown, Subject 1 had no changes in the clinical outcomes, while Subjects 2 and 3 had showed slight improvements in Six Minutes Walk test, Time Up-and-Go test, as well as a minor improvement in the Fugl-Meyer Lower Extremity Motor assessment score. Subject 2 also had a slight improvement in Barthel Index and Subject 3 showed a slight improvement in Functional Gait Index.

**Table 3:** Pre- and pos-assessment data

	Patient 1		Patient 2		Patient 3	
Clinical Outcomes	Pre	Post	Pre	Post	Pre	Post
Berg Balance Scale (0-56)	47	49	54	54	56	56
Functional Gait Index (0-30)	22	20	28	28	25	26
6 Min Walk Test (meters)	328	213	274	290	134	237
Time Up-and-Go Test (sec)	16.2	17.9	11.8	9.3	13.2	10.7
Fugl-Meyer LE (0-34)	25	26	27	28	25	26
Barthel Index ADL (0-20)	18	18	17	18	18	18

### H2 Usability

The time needed for donning and setup H2 was very short, less than 10 minutes elapsed from the time participants arrived before gait training was started. The doffing process was even faster, less than 2 minutes. No adverse effects were observed during training, including no skin irritation or redness, no sore spots, any pain or discomfort during or after training.

H2 also demonstrated significant autonomy in the context of battery power. A totally charged battery pack could run the exoskeleton for about 9 training sessions of an average of 40 minutes each session. Considering that in each session, a participant walked 30 minutes on average, H2 could run for more than 4 hours of continuous walking with a single battery charge. Also since the battery pack is detachable from the mechanical frame, it is very easy to replace an empty one with a fully charged battery pack.

The H2 tested was the first device built and thus, issues might have been expected. Remarkably, the H2 robot-assisted gait training was conducted without any major problems. Only minor technical issues occurred without impacting user’s safety and were easily fixed.

Lastly, patients participating in the study were very excited about training with the device. When asked to evaluate the ease to use of device in each session on a Likert scale, the average rating for the 3 patients in all 12 sessions were 7.2, where 0 indicates “extremely hard to use" and 10 indicates “extremely ease to use". The main positive feedback received from patients when training with H2 was: “the device is lightweight"; “wearing it is fast and simple"; “I can feel that it helps my knee flexion"; “it is more exciting walking overground with this device than my previous treadmill training with manual assistance" and “I wish I had access to this device when I was in the hospital for inpatient rehabilitation after my stroke". The main negative feedback received from patients was: “it felt weird at the first moment and took me some time to get used to it in my first training session, since I have never used a robotic device like this".

## Discussion

Here, we present the development and the first evidence for safety and usability of the H2 wearable robotic exoskeleton in the context of post-stroke gait rehabilitation. The main finding of this work is that the developed H2 exoskeleton provides a means for safe and intensive gait training in hemiparetic stroke survivors. Across 4 weeks of training in 3 stroke subjects, the H2 exoskeleton proved to be easy to use, with a fast donning and doffing process and was very well accepted by patients as a potential rehabilitation device.

Importantly, the results from this pilot clinical study indicate that the H2 operated in “assist-as-needed" control mode allows reshaping of the asymmetric, deviant hemiparetic gait in stroke survivors through a relatively short period of training. It is important to note that in most stroke victims, the lack of knee flexion during swing creates an abnormal compensatory movement in the hip, commonly known as hip hiking [49]. Also, most patients do not rely on their paretic leg, hence, they do not shift weight equally on both lower limbs during walking. This behavior creates an asymmetric gait pattern where the stance phase on the paretic leg is shorter than the unaffected leg. The gait assistance force field implemented in H2 guided patients in a correct gait pattern, creating a stance phase that is equal across both lower limbs and this could potentially prevent the compensatory hip hiking. As a result, while using H2 patients are being trained to the correct pattern of weight shifting between lower limbs and knee flexion, which can further contribute to the H2’s therapeutic benefit.

Actuation at the ankle was another important aspect of H2 design. During training it helped avoid foot drop and could help patients to work on dorsiflexion movements. The H2’s control algorithm, therefore, helps these patients relearn a symmetric gait pattern across both lower limbs by providing assistance as needed at the appropriate limb segments and joints. Importantly, the ability to perform this training in a functional context such as over ground walking is of major clinical significance. Furthermore, it is very interesting to note that this training is stimulating and challenging even for the participant with chronic stroke (5 years ago). Coupled with the motivational component of training provided by a novel robotic gait training regimen, the H2 allows these participants to experience kinesthetic feedback of near-normal gait patterns in over ground walking. Since the 3 participants in this study were able to increase walking speed and distance across training sessions, it would appear that H2 robot-assisted training can potentially recruit extant neuroplasticity and promote improved motor control in these patients.

However, these findings must be considered with the caveat that this study is limited to a small subgroup of patients that is not representative of the entire stroke population and therefore, conclusions cannot be drawn regarding gait improvements after use of H2. Furthermore, as seen from the patient demographic data and functional outcome scores, the subgroup of stroke participants included in this study is also heterogeneous in terms of time at which H2-assisted training was provided with regards to their stroke onset as well as their individual functional impairments. This is an important factor to be considered as this population is very diverse, and therefore, no two patients are alike in terms of their impairments.

Therefore, it is critical that H2 robot-assisted training be personalized to each individual based on his/her needs. In this regard, further modifications can be implemented in the control algorithm to provide variable resistance once the user has reached a certain threshold in terms of torque generation and/or joint angular position/velocity. This will help ensure progressive, adaptive changes to the training regime and is a clinically significant issue that warrants further investigation.

Notably, the modular design of the H2 is particularly relevant for stroke rehabilitation. Since various segments of the device can be used independently, H2 offers promising means of using unilateral Hip-Knee-Ankle, Knee-Ankle or only Ankle versions of the device, customizing treatment protocols to each patient’s specific needs. These questions need to be addressed in future research, in order to help develop optimal control algorithms to use these modular components of the H2 for individualized rehabilitation. Similarly, appropriate intervention durations, and frequency of training i.e., “dosing schedules" are still not well established for such wearable robotic rehabilitation protocols, which also needs to be examined in careful detail in further clinical investigations. Furthermore, in order to fully utilize the functionality of the lightweight wearable H2 device, future training protocols can also include other functional tasks such as sit-to-stand, stand-to-sit and stair climbing.

As seen (Table 3), lack of major changes in clinical outcomes precludes any conclusions about functional improvements when training with H2 in this study. We believe this is primarily because while the participants in this study had qualitative gait asymmetries and impairments, this is not captured by the granularity of the standard clinical outcome measures. Further, in some of the items such Berg’s Balance Scale and Functional Gait Index, if participants achieve scores closer to the ceiling, it is impossible to track any further qualitative improvement using those items. This brings to light the importance of developing novel metrics or outcome measures that are sensitive and capable of tracking behavioral changes quantitatively and qualitatively in robotic rehabilitation paradigms. However, it is important to study the relationship of these novel metrics to standard clinical outcomes, in order to describe the functional domain that is being assessed. Our research efforts are currently directed towards this, as it is a very important factor in the clinical context.

Finally, the factors discussed above such as inadequate “dosing" in terms of frequency and duration of training may have prevented sufficiently progressing treatment for each participant based on their functional levels. These questions need to be addressed in a clinical investigation with a larger population, along with comparison of H2 robot-assisted training to conventional physical rehabilitation regimes. Our future work, therefore, is focusing on a controlled clinical study in a larger sample of participants with stroke.

## Conclusion

In summary, this work presents the development and evaluation of H2, a novel lower limb robotic exoskeleton for rehabilitation of stroke survivors. This device is lightweight and battery-powered, thereby allowing for gait training in functional contexts such as overground walking in comparison to more traditional tethered or treadmill-based robotic rehabilitation devices. Further, the control of H2 is based on a custom assist-as-needed algorithm that creates a force field along a desired trajectory, proportionally applying torque only when patient deviates from the pre-programmed correct pattern. This force field control, therefore, can help restore a symmetric gait pattern in hemiparetic stroke survivors, by assisting only the segments that need it and possibly preventing undesired compensatory movement patterns, such as hip hiking.

Additionally, a customized mobile-based user interface allows the therapist to personalize and adjust the maximum allowed deviation from the reference based on a specific patient’s condition. Finally, we also present early findings from a clinical evaluation of the H2 for gait rehabilitation in 3 participants with post-stroke hemiparesis. Participants showed adaptive improvements in their gait trajectories across the training sessions over 5 weeks. These results are encouraging and provide the first evidence for safety and feasibility of using the H2 for functional gait training in stroke patients. Our future work aims to evaluate the therapeutic benefits of active training with the exoskeleton in restoring gait function in a larger population of stroke patients.

In summary, the developed H2 device opens up future research avenues to study methods to optimize rehabilitation protocols that can be customized for individuals with gait impairments following neurological injuries and with the capacity to deliver high dosage and high intensity therapies. Taken together, these advances can have a huge clinical impact by helping accelerate recovery and improve functional independence and quality of life in these patients.
